# Glycoform-independent prion conversion by highly efficient, cell-based, protein misfolding cyclic amplification

**DOI:** 10.1038/srep29116

**Published:** 2016-07-07

**Authors:** Mohammed Moudjou, Jérôme Chapuis, Mériem Mekrouti, Fabienne Reine, Laetitia Herzog, Pierre Sibille, Hubert Laude, Didier Vilette, Olivier Andréoletti, Human Rezaei, Michel Dron, Vincent Béringue

**Affiliations:** 1 VIM, INRA, Université Paris-Saclay, 78350, Jouy-en-Josas, France; 2 IHAP, INRA, Ecole Nationale Vétérinaire de Toulouse, 31000, Toulouse, France

## Abstract

Prions are formed of misfolded assemblies (PrP^Sc^) of the variably N-glycosylated cellular prion protein (PrP^C^). In infected species, prions replicate by seeding the conversion and polymerization of host PrP^C^. Distinct prion strains can be recognized, exhibiting defined PrP^Sc^ biochemical properties such as the glycotype and specific biological traits. While strain information is encoded within the conformation of PrP^Sc^ assemblies, the storage of the structural information and the molecular requirements for self-perpetuation remain uncertain. Here, we investigated the specific role of PrP^C^ glycosylation status. First, we developed an efficient protein misfolding cyclic amplification method using cells expressing the PrP^C^ species of interest as substrate. Applying the technique to PrP^C^ glycosylation mutants expressing cells revealed that neither PrP^C^ nor PrP^Sc^ glycoform stoichiometry was instrumental to PrP^Sc^ formation and strainness perpetuation. Our study supports the view that strain properties, including PrP^Sc^ glycotype are enciphered within PrP^Sc^ structural backbone, not in the attached glycans.

Prion phenotype results from the conformational change of specific amyloidogenic proteins. This change is based on the self-sustained transfer of a structural information from a protein conformer in the prion state to the same protein in the non-prion conformation, presumably through a seeding-polymerization process. Initially formulated to explain prion diseases pathogenesis in human and animals, the prion concept has gained wider relevance in the regulation of diverse biological process and in the progression of other neurodegenerative disorders such as Alzheimer and Parkinson diseases^1^,^2^,^3^. Mammalian prions are primarily formed of macromolecular assemblies of PrP^Sc^, a misfolded, ß-sheet enriched form of the ubiquitously expressed, α-helix rich, host-encoded prion glycoprotein PrP^C^. Within defined host species, PrP^C^ can be transconformed in many prion variants or strains, differing in their PrP^Sc^ conformation at the level of the tertiary and/quaternary structure and in their biological properties^4^,^5^,^6^,^7^. In particular, prions maintain strain-specific stoichiometric ratios of PrP^Sc^ glycoforms on serial passaging in the same host species^8^,^9^, leading to the view that glycans may somehow participate to prion strain information encoding. Consistently, transgenic modelling suggested that PrP^C^ glycosylation status influenced the efficacy of intra- and cross-species transmission of prions^10^,^11^,^12^ and prion strain properties^13^. However, such studies remained difficult to interpret, given that point mutations inserted to prevent N-linked glycosylation or altered trafficking of the mutant PrP^C^ rather than N-glycans removal may be the primary cause for the observed alterations in the propagation of prions (see ref. 14 and references herein). The intrinsic convertibility of PrP^C^ glycosylation mutants into PrP^Sc^ and the role of attached glycans in prion strainness remain thus an open question.

While the molecular mechanisms and the cellular factors potentially involved in PrP^Sc^ formation remain largely undefined, PrP^C^ is convertible into PrP^Sc^ in a test tube after adjunction of minute amounts of PrP^Sc^ seeds by a technique designated protein misfolding cyclic amplification (PMCA)^15^. PMCA increases the ability of PrP^Sc^ to template the conversion of PrP^C^ by repetitive cycles of incubation and sonication. As the main source of PrP^C^ substrate, most of the proprietary PMCA protocols are using brain homogenate from susceptible animals or transgenic mouse models expressing the PrP^C^ of interest. The sensitivity achieved by PMCA allows amplification of subinfectious levels of PrP^Sc^ in biological samples such as blood, urine, faeces or cerebrospinal fluid of human and animals infected with prions^16^,^17^,^18^,^19^,^20^. PMCA products or ‘amplicons’ are truly infectious and generally exhibit the same strain properties as the PrP^Sc^ seeds^21^,^22^,^23^,^24^. A limited number of experiments have been performed by replacing brain substrate with cell substrate^25^,^26^,^27^,^28^ despite the availability of a number of cell models expressing PrP^C^ from different species, and permissive to prions (for review^29^). These cell-based PMCA assays generally yielded either low PrP conversion rates or unsatisfying sensitivity to be applied routinely in high throughput protocols. While using brain material is not a limiting step for routine use of PMCA, addressing the contribution to the prion conversion process of certain PrP^C^ polymorphisms or mutations or post-translational modifications, such as glycosylation becomes an issue with this technique when suitable transgenic mouse models are not available.

In the present study, we adapted the miniaturized-bead PMCA (mb-PMCA) protocol^22^ to the use, as PrP^C^ substrate, of cell lysates from RK13 cell lines^30^ expressing PrP^C^ from different species or with point mutation. We report highly efficient amplification of scrapie, hamster, human and to a lesser extent of mouse-adapted prion strains. We next addressed the question of the prion convertibility of several ovine PrP glycosylation mutants defective in glycosylation at either sites of PrP or at both sites^14^. At variance with earlier reports using cell^14^,^31^ or transgenic mouse^11^ modelling, PrP^C^ glycosylation mutants were converted as efficiently as wild-type PrP^C^ into *bona fide* prions by PMCA with two unrelated prion strains. Our study also reveals that interactions between defined stoichiometric ratios of neither PrP^C^ nor PrP^Sc^ glycoforms are key to PrP^Sc^ formation, leading to the view that strain-specific glycotype is enciphered within PrP backbone. In ethical and practical terms, the development of a highly sensitive cell lysate-based PMCA will allow reduction and even bypassing the use of animal tissues.

## Results

### Cell-based mb-PMCA efficiently amplifies prions

**Figure 1:**
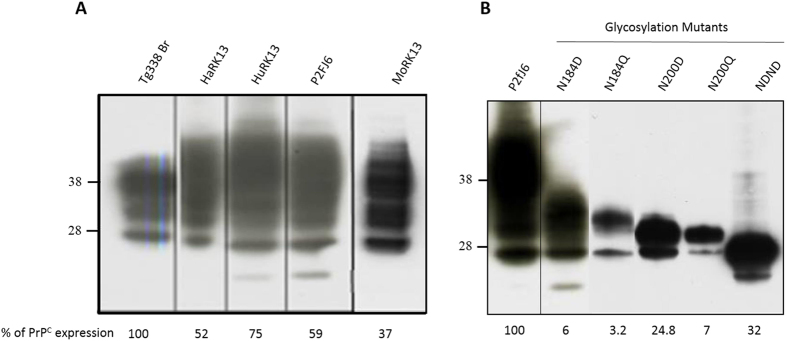
Electrophoretic profiles and expression levels of PrP^C^ in the different RK13 cell lines used. (**A**) RK13 cell lines expressing hamster (HaRK13), human (HuRK13), sheep (P2FJ6 clone) and mouse (MoRK13) wild-type PrP and (**B**) the different ovine PrP glycosylation mutants (as referenced in Table 2) were probed for the presence of PrP^C^ by western blotting (SAF34 anti-PrP monoclonal antibody). Brain lysate of ovine PrP tg338 mice (**A**) or cell lysates from P2FJ6 clone (**B**) were used to determine the relative expression levels in the different cell lines. Migration size of standard molecular mass marker (kDa) is indicated on the left.

Previously, we developed a so-called mb-PCMCA procedure allowing efficient amplification of prions from different species in a round of 48 hours and in a micro-plate format^18^,^22^,^32^. The experimental conditions were primarily established with brain material from transgenic tg338 mice overexpressing ovine PrP^C^ and the 127S scrapie strain, a prototypal ‘fast’ strain killing tg338 mice within 2 months^33^. To progressively replace tg338 brain substrate by cell substrate, we used rabbit kidney epithelial RK13 cells expressing constitutively the VRQ allele of ovine PrP^C^ (P2FJ6 clone), and susceptible to 127S prions^32^,^34^. At the same protein concentration, these cells express approximately 2/3 of PrP^C^ levels compared with tg338 mouse brain, and the PrP^C^ glycoform pattern is cell-specific (Fig. 1A and refs 30,35). The cell-based mb-PMCA (Cell-mb-PMCA) procedure was performed by seeding serial 10-fold dilutions of 127S-infected brain homogenate in P2FJ6 cell lysates, and running 96 incubation/sonication cycles for a round of 48 h in 96-well microplates. The amplicons were treated with proteinase K (PK) to eliminate PrP^C^ before detection of PK-resistant PrP^Sc^ (PrP^res^) by Western-blotting.

**Figure 2:**
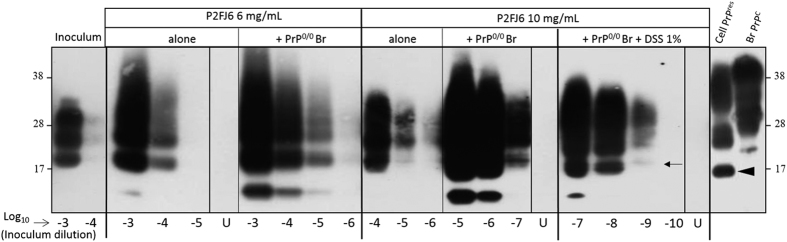
Endpoint titration of 127S prions by Cell lysate based mb-PMCA supplemented with PrP knockout brain. P2FJ6 cell lysates were prepared at two protein concentrations and used either alone or mixed (1:1) with 10% PrP^0/0^ mouse brain (Br) lysate in the absence and the presence of 1% Dextran sulphate sodium (DSS), as indicated. The lysates were then used as PMCA substrate to amplify serial 10-fold dilutions of brain homogenate from tg338 mice infected with 127S prions. Each dilution was directly analysed by Western blotting for PrP^res^ content (Sha31 antibody) after proteinase K treatment. For comparison purposes, the first two lanes illustrate PrP^res^ content in non-amplified products (10^−3^ and 10^−4^ dilutions); the last two lanes PrP^res^ and PrP^C^ electrophoretic profiles in 127S infected P2FJ6 cell line (Cell PrP^res^) and normal tg338 mouse brain lysates (Br PrP^C^), respectively. Lanes U correspond to unseeded lysates ran on the same microplate. Note the difference in unglysosylated PrP^res^ molecular mass between Cell-PMCA generated products (small arrow) and cell-passaged prions (arrowhead). *Low size PrP^res^ fragments.

Cell-mb-PMCA efficiency to amplify 127S prions was tightly dependent of the total protein concentration in the cell lysate. At the concentration of 2 mg/mL, there was no significant amplification of 127S seeds (not shown). At 6 mg/mL, PrP^res^ was detected in reaction mixtures seeded with 10^4^-fold diluted 127S brain homogenate. Further concentration of the cell lysate to 10 mg/mL led to detection of PrP^res^ from reaction mixtures seeded with 10^5^–10^6^-fold diluted inoculum (Fig. 2). Supplementation of the 10 mg/mL cell lysate with brain homogenate from PrP^0/0^ mice (1:1 ratio) allowed detection of PrP^res^ in 127S brain homogenate diluted up to 10^7^-fold (Fig. 2). PMCA performed with untransfected parental RK13 cells resulted in no detectable amplification of PrP^res^ (Fig. S1), consistent with the non-detection of endogenous rabbit PrP^C^ in these cells^30^ and the poor convertibility of rabbit PrP^C^ by 127S-like prions^36^.

Various molecules reportedly enhance prion conversion and amplification, in a strain-dependent manner^37^,^38^,^39^,^40^,^41^,^42^,^43^. We examined whether addition of negatively charged molecules, such as dextran sulfate (DSS) would further improve Cell-mb-PMCA sensitivity. Addition of 1% DSS (>500 kDa) led to the amplification of the 127S strain up to 10^−9^ dilution, i.e. approx. 10–100-fold less the sensitivity routinely obtained with tg338 brain as substrate^22^. All the unseeded samples were negative in these experiments (Fig. 2, lanes U). The conversion yield of PrP^C^ present in the cell lysate, as examined after thermolysin treatment of the amplified products^22^ was ≤30% (data not shown), as previously observed with tg338 mouse brain material^22^.

Compared to brain PrP^res^ (Fig. 2, lanes Inoculum), the PrP^res^ glycoprofile of the amplified product was cell-specific, consistent with the differences in PrP^C^ glycans content between brain and non-neuronal cultured cell models^30^,^35^. Furthermore, the cell-mb-PMCA amplicon exhibited a higher molecular mass (~2 kDa) than that of PrP^res^ accumulating in infected P2FJ6/Rov cell cultures (Fig. 2, compare with Cell PrP^res^ lane). In Rov cells, biosynthetized PrP^Sc^ is naturally cleaved by cathepsin proteases, to produce the so-called C2 fragment. This fragment is more truncated than the PK-resistant core of 127S PrP^Sc ^35. Conceivably, interactions between PrP^Sc^ and cathepsin proteases may not occur during Cell-mb-PMCA, due to disruption of endolysosomial vesicles by the detergent used in the PMCA lysis buffer.

We next determined whether the infectivity of the amplified products generated using cell lysates would correlate with the efficacy of amplification. The amplicons obtained with the 10^−7^ 127S seed with 10 mg/mL cell lysates supplemented with PrP^0/0^ brain, in the presence or absence of 1% DSS, were tenfold diluted up to the 10^−7^ dilution and immediately inoculated intracerebrally to reporter tg338 mice (Table 1). Mice inoculated with unseeded controls did not develop any clinical disease and were euthanized healthy at 240 days post-inoculation. A 100% attack rate was observed with the cell-generated amplicons diluted up to 10^−4^ fold. At the 10^−5^ dilution, 1/5 (−DSS) and 2/5 (+DSS) tg338 mice were infected. At the 10^−6^ and 10^−7^ dilution, none of the mice developed the disease and were euthanized healthy. There was thus no significant impact of adding DSS to the PMCA reaction on the infectivity of the amplified products. Collectively, these data indicate that the cell-generated amplicons were 100-fold less infectious than the brain-generated amplicons (Table 1 and ref. 22). This value was consistent with the difference of amplification efficiency observed between cell and brain lysates.

**Figure 3:**
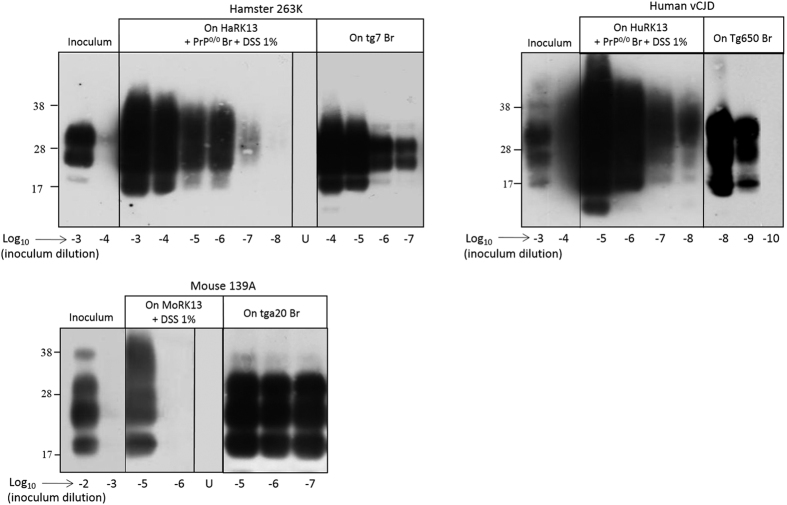
Comparison of the efficacy of brain-based or cell-based PMCA to amplify hamster, vCJD and mouse prions. Lysates from RK13 cell lines expressing hamster, human and mouse PrP^C^ (supplemented with 1% DSS and eventually PrP^0/0^ brain (Br)) or brain homogenates from hamster PrP (tg7), human PrP (tg650) and mouse PrP (tga20) mice were seeded with serial dilutions of brain homogenates containing hamster 263K prions, human vCJD prions or mouse 139A prions and submitted to a single round (263K, vCJD) or 2 rounds (139A) of PMCA. Unamplified inoculums (two first lanes of each panel), unseeded controls (lanes U) and the amplified samples were digested with PK before western blotting (Sha31 antibody) analysis for PrP^res^ content.

We next examined whether the Cell-mb-PMCA protocol (10 mg/mL protein concentration, addition of 1% DSS and PrP^0/0^ brain) would amplify prion from other species. We seeded RK13 cell lysates expressing hamster (HaRK13), human (methionine at codon 129, HuRK13) or mouse (MoRK13) PrP^C^ (Fig. 1^44^ and unpublished data) with serial dilutions of 263K, vCJD and 139A prions, respectively and compared the sensitivity achieved with that obtained with transgenic mouse brain as substrate. The results are summarized in Fig. 3, which is representative of more than 4 independent experiments. PrP^res^ from hamster 263 K and human vCJD prions was amplified from 10^−7^- and 10^−8^-diluted input seeds by using HaRK13 and HuRK13 cell substrate, respectively. This sensitivity was close to that obtained with transgenic mouse brain (Fig. 3 and ref. 22). In contrast, 139A prions were less efficiently amplified, as two PMCA rounds without PrP^0/0^ brain supplementation were necessary for PrP^res^ detection from the 10^−5^ dilution, compared to the 10^−7^ dilution amplified in one round with tga20 brain lysate (Fig. 3 and ref. 22). More sensitive cell clones expressing higher levels of mouse PrP^C^ (Fig. 1) or Mo RK13 cell-specific conditions are to be found to improve the amplification of 139A prions.

Collectively, our data indicate that Cell-based PMCA, as mouse brain based mb-PMCA^22^, is a versatile protocol, allowing amplification of minutes amounts of prions from different species, including human.

### Cell lysates with high protein concentration avoid the use of PrP knock-out mouse brain material and DSS for efficient prion amplification

**Figure 4:**
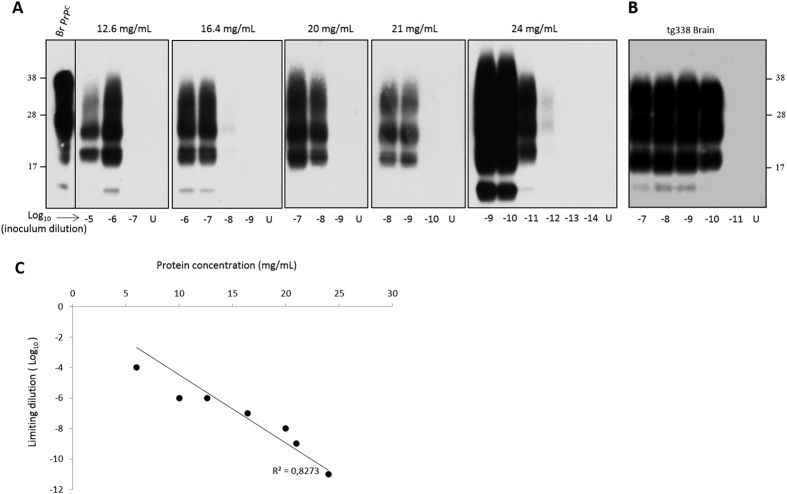
Highly efficient amplification of 127S prions using concentrated cell lysate alone by a single round of Cell-mb-PMCA. (**A**) P2FJ6 cell lysates were concentrated with regard to the total protein concentration and were directly seeded with serial 10-fold dilutions of tg338 brain homogenate containing 127S prions for a single round Cell-mb-PMCA. (**B**) In parallel, the same dilutions were submitted to mb-PMCA by using tg338 brain homogenate as substrate. Unseeded controls (lanes U) and the amplified samples were digested with PK before western blotting analysis for PrP^res^ content (Sha31 antibody). The first lane contains undigested normal tg338 mouse brain. (**C**) Regression analysis of 127S endpoint dilution achieved by cell-mb-PMCA versus total protein concentration in the P2FJ6 cell lysate.

The positive correlation between the protein concentration in the cell lysate used as substrate and the PMCA sensitivity to detect 127S prions led us to reason that increasing further the total protein concentration over 10 mg/mL in the cell lysates might allow efficient prion amplification without additives. To obtain highly concentrated cell lysates, we cultivated P2FJ6 cells in multilayer preparative flasks. The cell lysates were then used ‘crude’ in Cell-mb-PMCA reactions. As shown in Fig. 4A, concentrating cell lysates from 12 mg/mL to 24 mg/mL increased the sensitivity of 127S detection by 5 Log_10_. At that concentration, the sensitivity achieved was similar or even higher than that obtained with tg338 brain substrate run in the same micro-plate (Fig. 4B). Reporting the limiting dilution achieved to the total protein concentration in the cell lysate showed a strong correlation between total protein concentration and the efficacy of PMCA amplification (Fig. 4C). Of note, a 10% brain homogenate would provide a 10–12 mg/mL protein concentration. Thus, the use of highly concentrated cell lysate with regard to protein content allows amplifying minute amounts of 127S prions in a brain-free context.

### Efficient Cell-based mb-PMCA conversion of ovine PrP glycosylation mutants by two distinct prion strains

**Figure 5:**
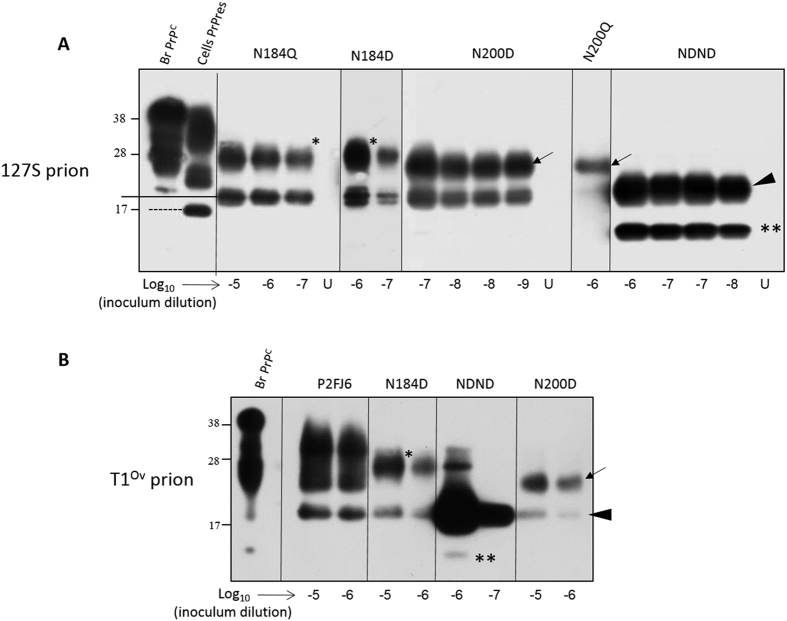
Efficient conversion of PrP^C^ glycosylation mutants by two distinct prions in Cell-mb-PMCA reactions. Lysates from RK13 cells expressing ovine PrP^C^ mutated on the first N-glycosylation site at residue 184 (N184Q, N184D), on the second glycosylation site at residue 200 (N200D, N200Q) or at both residue (NDND) glycosylation sites were mixed with PrP^0/0^ brain lysate (1:1 dilution), seeded with serial 10-fold dilutions of tg338 brain homogenate containing either 127S (**A**) or T1^Ov^ (**B**) prions and submitted to 2 rounds of Cell-mb-PMCA. Lanes U contains unseeded controls. All samples were PK-treated before western blot analysis (Sha31 antibody). For comparison, the electrophoretic profiles of tg338 brain PrP^C^ (lane Br. PrP^C^) and P2FJ6 PrP^res^ (lane Cell PrP^res^) are shown. To facilitate the interpretation of the western blot, PrP^res^ monoglycosylated at residue 200 or 184 are indicated by asterisks and arrows, respectively. Full-length unglycosylated PrP^res^ is indicated by an arrowhead. **Indicates low size PrP^res^ fragments, highly abundant following conversion of unglycosylated PrP^C^ by 127S but not by T1^Ov^ prions.

PrP^C^ has two variably occupied glycosylation sites at amino acid N184 (site 1) and N200 (site 2) (ovine PrP sequence numbering). Modelling in RK13 cells previously suggested that unglycosylated double PrP mutant failed to be converted by 127S prions, even after being properly expressed at the cell surface by an ectopic glycosylation site in the N-terminus of PrP^C ^14. Prion convertibility of monoglycosylated mutant was site-dependent with mutants at site 2 all being convertible and mutants at site 1 being convertible only when the N184D amino acid substitution was performed. These negative results opened the possibility that some mutants were intrinsically not convertible into prions, due to point mutation or to N-glycans removal. We examined this possibility by seeding the different cell lysate mutants (Fig. 5 and Table 2) with serially diluted seeds of 127S prions and running Cell-mb-PMCA (10 mg/mL cell lysate supplemented with 10% PrP^0/0^ brain homogenate, no DSS).

Figure 1B illustrates PrP^C^ electrophoretic pattern and expression level in the different glycosylation mutant cell lysates relative to the wild type P2FJ6 cells. Overall, PrP^C^ expression level in the different PrP glycosylation mutants was low, ranging from ~3% (N184Q) to 32% (NDND double mutant). All the PrP^C^ glycosylation mutants were converted into PrP^Sc^ by Cell-mb-PMCA. In one round, the limiting dilution of the 127S input seed established at 10^−5^ for N184Q and N200Q and 10^−6^ for N200D and NDND mutants. Despite low expression levels of mutant PrP^C^ in the cell lysates, the sensitivity achieved was thus only 100- (N184Q) and 10-fold less (N200D, NDND) than that observed by using wild-type cell lysates. After a second round (Fig. 5A), the limiting dilution established between 10^−7^ and 10^−9^ for all but the N200Q mutants, which established at 10^−6^. Taken together, these results demonstrate that unglycosylated and monoglycosylated PrP^C^ mutants are intrinsically convertible into PrP^Sc^ by 127S prions, independently of their non-convertibility once expressed in RK13 cells.

We next examined whether the absence of PrP^C^ glycosylation requirement for *in vitro* prion conversion would apply to another prion strain designated T1^Ov^, and obtained after adaption to tg338 mice of prions responsible for a rare cortical, MM2 form of sporadic Creutzfeldt-Jakob disease^45^. T1^Ov^ prions have no strain properties in common with 127S prions but share similar efficacy to be amplified by PMCA using tg338 brain as substrate^45^. In two rounds, the limiting dilution of the T1^Ov^ input seed established at 10^−6^ for all the N184D and N200D mutants, as for wild-type PrP, and 10^−7^ for the NDND mutant (Fig. 5B). It can be noted that the proportion of low-size PrP^res^ fragments in the lowly-glycosylated amplicons markedly differed between the two strains (Fig. 5), further differentiating the two agents.

### Cell-based mb-PMCA lowly glycosylated prions are highly infectious

We finally addressed whether the monoglycosylated or unglycosylated PrP^Sc^ products generated by glycosylation mutant Cell mb-PMCA were infectious and retained strain-specific biochemical and neuropathological properties. Amplicons generated from reaction mixtures seeded with 10^−7^ 127S brain material, -and amplified over 2 rounds to exclude any residual infectivity of the input seed-, were inoculated by intracerebral route to reporter tg338 mice. As shown in Table 2, the amplified products generated with the glycosylation mutants induced disease in mice with similar efficacy as the products generated on the wild-type PrP cell substrate. Mean incubation time to disease was the shortest after inoculation of the non-glycosylated amplicons (68 days) compared to wild-type generated amplicons (70 days). N184Q-derived amplicons were the less efficient, inducing disease in 77 days. Reporting the incubation time values to 127S dose-response curve^34^ and quantifying the amount of PrP^res^ in the amplicons allowed calculating specific infectivity values, that is the amount of infectivity per molecule of PrP^res^ generated by the PMCA reaction. Assuming a straight correlation between PrP^res^ content in the PMCA amplicons and infectivity, the specific infectivity per unit PrP^res^ appeared 10 to 80-fold higher for the lowly –glycosylated amplicons than for the wild-type amplicons (Fig. S2).

**Figure 6:**
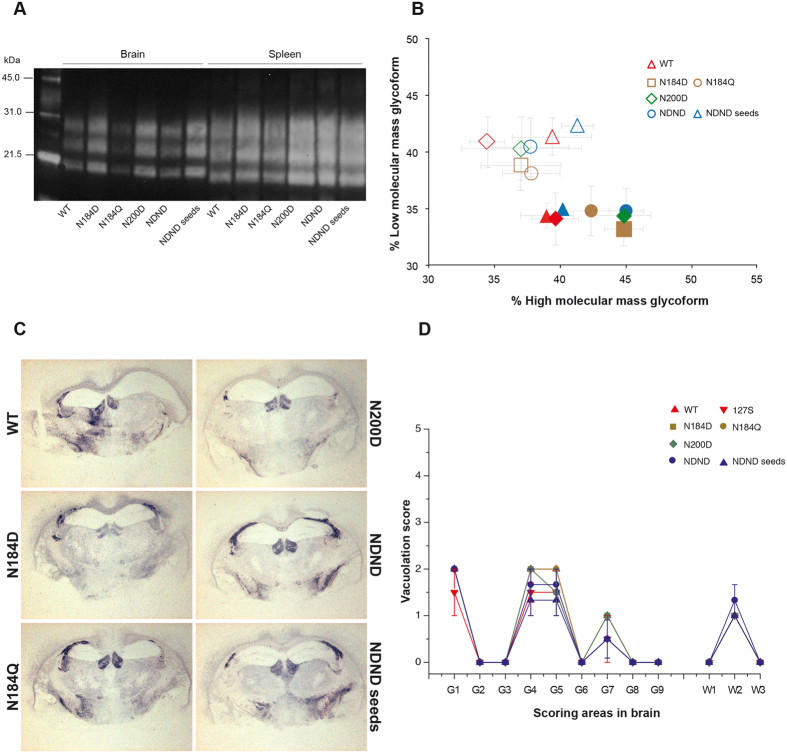
Strain properties of Cell-mb-PMCA lowly-glycosylated 127S prions in tg338 mice. Lysates from RK13 cells expressing wild-type (WT) ovine PrP^C^ or ovine PrP^C^ mutated on the first N-glycosylation site at residue 184 (N184D, N184Q), on the second glycosylation site at residue 200 (N200D) or at both residue (NDND) glycosylation sites were mixed with PrP^0/0^ brain lysate (1:1 dilution), seeded with serial 10-fold dilutions of tg338 brain homogenate containing 127S prions and submitted to 2 rounds of Cell-mb-PMCA before inoculation to tg338 mice. Seeds generated from NDND cells were also submitted to another round of PMCA using tg338 mouse brain as substrate (wild type brain PrP^C^). The amplicon obtained at 10^−8^ dilution was then used for inoculation (NDND seeds). (**A**) PrP^res^ banding pattern and (**B**) ratios of high- and low-molecular mass PrP^res^ glycoforms in the brain (filled symbol) and spleen (open symbol) tissue of tg338 mice inoculated with Cell-mb-PMCA products. (**C**) Neuroanatomical distribution of PrP^res^ in tg338 mice inoculated with the Cell-mb-PMCA products. Representative histoblot (12F10 antibody) of brain coronal section (hippocampus level). Deposition in standardized anterio-posterior sections can be vizualized in Supplementary Figure S1. (**D**) Distribution of vacuolar degeneration (lesion profile) in tg338 mouse brain inoculated with the Cell-mb-PMCA products, as above. The intensity of vacuolation was scored as means standard errors of the means (error bars) in standard gray (G1 to G9) and white (W1 to W3) matter areas. These areas are as follows: G1, dorsal medulla; G2, cerebellar cortex; G3, superior colliculus; G4, hypothalamus; G5, medial thalamus; G6, hippocampus; G7, septum; G8, medial cerebral cortex at the level of the thalamus; G9, medial cerebral cortex at the level of the septum; W1, cerebellar white matter; W2, white matter of the mesencephalic tegmentum; and W3, pyramidal tract.

Remarkably, PrP^res^ electrophoretic pattern in brain and spleen tissue (Fig. 6A,B), and neuroanatomical distribution of PrP^res^ (Figs 6C and S3) and of vacuolar degeneration (Fig. 6D) in the reporter tg338 mice inoculated with unglycosylated or monoglycosylated 127S amplicons was reminiscent of 127S prions, passaged (Fig. 6) or not^19^,^33^,^46^,^47^ by Cell-mb-PMCA. Prominent PrP^res^ deposition in the lateral hypothalamic area, in the corpus callosum, in the habenula (Fig. 6C) in the raphe nuclei of the brain stem (Fig. S3), and marked vacuolar degeneration in the dorsal medulla, hypothalamus and white matter of the mesencephalic tegmentum (Fig. 6D) were typical of 127S prions^33^. PrP^res^ staining and vacuolation in the affected brain regions were sometimes less intense, as observed with infection of diluted 127S-infected tg338 brain homogenate^33^ or on reisolation of 127S prions to tg338 mice^19^,^46^,^47^.

**Figure 7:**
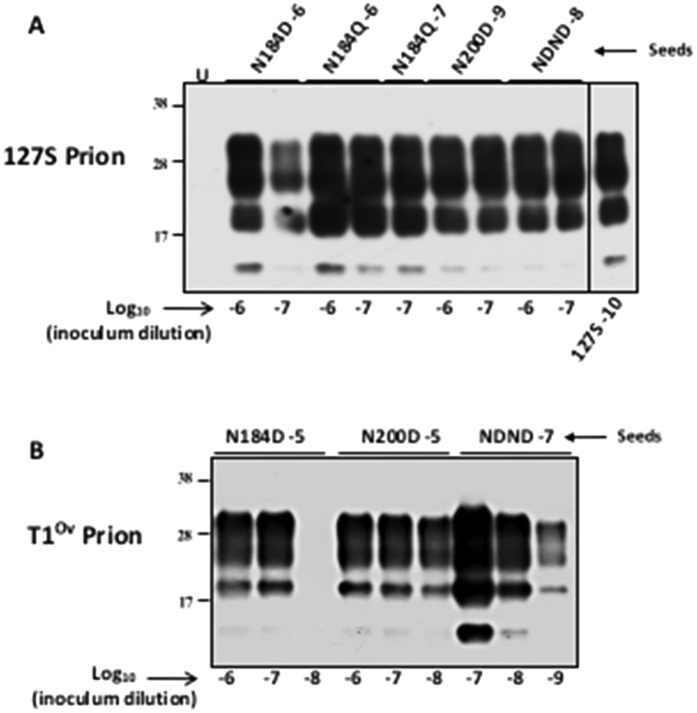
Converting activity of PMCA-generated lowly glycosylated prions. Serial dilutions of lowly-glycosylated 127S (**A**) and T1^Ov^ (**B**) prions generated by cell-mb-PMCA (at the indicated Log_10_ dilution) were submitted to mb-PMCA using tg338 mouse brain as substrate. All the samples were PK-treated before western blot analyses. Amplification of 127S prions is shown for comparison (**A**). U: unseeded control.

To further ascertain that PMCA-generated lowly glycosylated amplicons were good convertors of wild-type ovine PrP^VRQ^, 127S and T1^Ov^ amplicons were submitted to mb-PMCA using tg338 mouse brain as substrate. The seeding activity of 127S and T1^Ov^ amplicons was observed up to the 10^−7^ and 10^−9^ dilution (Fig. 7A,B), as with 127S amplicons generated from wild-type cells (Fig. 2) or with T1^Ov^ prions from brains of terminally sick tg338 mice^45^, respectively.

One of the 127S PMCA products generated with unglycosylated PrP^res^ seed (10^−8^) was inoculated to reporter tg338 mice to further confirm efficient conversion and maintaining of strain properties. The survival time of the mice, PrP^res^ electrophoretic pattern in brain and spleen tissue, and PrP^res^/vacuolar deposition patterns in the brain were all consistent with the generation of (highly) infectious 127S prions (Table 2, Figs 6 and S3).

Collectively, these data indicate that 127S prion strain properties and T1^Ov^ seeding capacity were essentially conserved despite intermediate replication on lowly glycosylated PrP^C^ species. This lends support to the view that glycans do not play a major role in prion replication dynamics and strain biological properties.

## Discussion

Following our simplification of the PMCA method, we now report that cell lysate expressing PrP^C^ can conveniently replace brain substrate from PrP transgenic mice to achieve efficient amplification of prions from different species. Highly concentrated cell lysate may permit amplification at ‘maximal’ levels without the need to supplement the reaction mixture with PrP knockout brain substrate. Applying the cell-PMCA technique to a panel of cells expressing PrP^C^ glycosylation mutants and two prion strains demonstrates that unglycosylated and monoglycosylated mutants are intrinsically convertible and that PrP^C^ and/or PrP^Sc^ glycoforms stoichiometry does appear to alter neither PrP^Sc^ formation rate *in vitro* nor the biological properties of the formed prion (for at least the strain tested *in vivo*). PrP glycosylation may thus be dispensable to perpetuate prion strain information.

To approach with cell lysates the sensitivity obtained in one round of PMCA with the *ad hoc* transgenic mouse brain as substrate^22^, it was beneficial to use concentrated cell lysate with respect to total protein concentration, and to supplement it with PrP^0/0^ mouse brain lysate and 1% DSS. By using the 127S prions/ovine PrP^C^ combination, we further showed that the PMCA amplification threshold obtained with brain material could be reached by using highly concentrated cell lysate alone, at least with 127S prions. The respective contributions of DSS, PrP^0/0^ mouse brain and concentrated cell lysate to efficient prion conversion remain to be determined. Non-PrP^C^ cellular factors such as brain lipids or polyanionic scaffold molecules like sulphated glycans and RNA, which are known to improve PMCA^37^,^39^,^40^,^43^,^48^,^49^,^50^, may have been concentrated. The conditions used may also create a macromolecular crowded environment^51^ favouring highly efficient prion conversion. In ethical and practical terms, sensitive PMCA can thus be performed without requiring animal models.

Applying the cell-PMCA technique to a panel of cells expressing PrP^C^ glycosylation mutants demonstrates that unglycosylated and monoglycosylated PrP^C^ were intrinsically convertible by 127S prions, despite non convertibility in cultured Rov cells, even after apparent proper expression at the cell surface during biosynthesis^14^ or exposure to homologous prions (this study). The reasons for such discrepancies with regards to glycosylation requirements between cell-free and in-cell systems remain to be determined. Subtle alterations in the subcellular localisation/trafficking of the PrP^C^ mutants or different turnover could explain their non-conversion in the cell models. Folding and/or stability and/or resistance to clearance of the nascent PrP^Sc^ assemblies in Rov cells may necessitate incorporation of a certain threshold of di-glycosylated species.

The molecular basis for prion strain-specific glycopattern and its perpetuation over serial passage is poorly understood. Host PrP^C^ glycosylation has been reported to contribute to prion replication and to prion strain phenotype (reviews refs 52, 53, 54, 55). Both the infecting prions and the convertible PrP^C^ isoforms in the recipient host or tissue determine the glycopattern of each strain. Use of biochemically deglycosylated native PrP^C^ in PMCA reaction suggested that the stoichiometry of PrP^C^ glycoforms regulated prion formation in a strain-specific manner^56^. For example, formation of PrP^Sc^ on seeding with mouse RML or hamster Sc237 prions necessitates presence or absence of unglycosylated PrP^C^, respectively. Oppositely, the failure of PrP^C^ glycoform-specific antibodies^57^ to exert similar selectively towards PrP^Sc^ glycoforms^58^ lend to the proposal that the proportion of each PrP^C^ glycoform incorporated into nascent PrP^Sc^ assemblies was controlled by the defined glycoforms stoichiometry in the starting infectious seeds^52^,^58^. Indirectly supporting this hypothesis is the observation that the PrP^Sc^ glycoform ratio (for a given strain) is conserved whatever PrP^Sc^ aggregation size^32^,^34^. What information does bring our PMCA modeling with cell expressing PrP glycosylation mutants? First, the high conversion rate of the mono- and un-glycosylated PrP^C^ mutants relative to wild-type PrP^C^, despite expression at lowered levels in the cell lysates, would sustain the view that highly glycosylated PrP^C^ species interfere with prion conversion or that presence of N-linked glycans on the two sites in PrP^C^ cause steric hindrance for PrP^Sc^ formation or through stabilisation of the PrP^C^ native state. The latter point would be consistent with the observation that the structural sequence important for PrP oligomerization lies between the two N-glycosylation sites^59^ or just upstream^28^,^60^. Diglycosylated PrP^C^ species may thus have a dual role during the formation of PrP^Sc^ assemblies. Second, 127S prion seeds, which exhibit in tg338 mouse brain a determined PrP^res^ glycotype (45% diglycosylated species, 35% monoglycosylated, 20% unglycosylated^22^), convert indifferently unglycosylated and monoglycosylated PrP^C^ species alone or in combination. Because conversion is not monitored in real-time during PMCA reactions, a glycotypic preference may exist during the initial converting events but be trailed off within a 48 h round. It could be argued that in face of mono or unglycosylated PrP^C^ species, mono and unglycosylated PrP^Sc^ may have been preferentially amplified. However, when the opposite experiment was done, that is when PMCA-generated unglycosylated or monoglycosylated PrP^Sc^ seeds were submitted to PMCA in the presence of wild-type PrP^C^, the initial 127S PrP^Sc^ glycotype was fully restored, thus suggesting no preferential compatibility between PrP^C^ and PrP^Sc^ with regard to the occupancy of the glycosylation sites. The same observations were made with a Creutzfeldt-Jakob disease derived prion strain designated T1^Ov^, thus indicating that the non-requirement of PrP glycosylation for prion conversion is not limited to one peculiar strain.

We finally show that monoglycosylated and unglycosylated 127S amplicons share similar strain properties as normally glycosylated 127S prions in tg338 mice, including the PrP^res^ glycotype in the brain of the mice. Collectively, we can conclude that a defined stoichiometry of PrP^Sc^ glycoforms and of PrP^C^ glycoforms is not necessary for efficient conversion by PMCA, and to dictate strain-specific properties, at least for 127S prions. Prion strain properties, including the glycotype stoichiometry of PrP^Sc^, may thus be solely enciphered within PrP^Sc^ structural backbone or within the way PrP^Sc^ molecules do assemble.

## Methods

### Ethics Statement

All animal experiments were carried out in accordance with the European Union directive 2010/63 and were approved by COMETHEA, the local ethics committee of the authors’ institution (permit number 12/034).

### Transgenic mice and Prion strains

The transgenic lines (tg338, tg7, tga20 and tg650 lines) and prions (127S, T1^Ov^, 139A, 263K and vCJD) have been previously described^22^,^33^,^34^,^45^. Pools of prion-sick mouse brains were prepared as 20% (wt/vol) homogenate in 5% glucose by use of tissue homogenizer (Precellys 24 Ribolyzer, Ozyme, Bertin technologies, France). The homogenate was diluted half to 10% in PMCA buffer (see below) to obtain the 10^−1^ dilution of the inoculum and stored at −80 °C. The Zürich I mouse line on an Sv129 mouse background was used as PrP^0/0^ line^61^.

### Cell culture

The Rabbit kidney epithelial RK13 cell line was used to establish cells expressing sheep (Rov9, P2FJ6 and glycosylation mutants), hamster (HaRK13), human (HuRK13) and mouse PrP (MoRK13). Rov9, P2FJ6 clones, cells expressing glycosylation mutants and MoRK13 cells have been described previously^30^,^32^,^34^,^44^. The open reading frame of hamster and human PrP^C^ was PCR amplified from Syrian hamster and human (Met 129 allele) genomic DNA, cloned into pBluescript plasmid, before subcloning in the pTRE and pCDNA plasmids (Clontech), respectively. After sequencing, each plasmid was introduced into RK13 cells as described previously^30^, and puromycin-resistant cell clones were selected for doxycycline-inducible and constitutive expression of PrP^C^, respectively. Cells were cultivated at 37 °C in 5% CO_2_ in Opti-MEM (Gibco) supplemented with 10% foetal calf serum and 0.1% penicillin and streptomycin. Cells were passaged once a week at a ¼ dilution. For production of large amount of concentrated cell lysates, cells were cultured in 2 or 4 layers of multilayer cell culture flasks (Thermo-Scientific Nunc).

### Preparation of cell lysate for PMCA

Cultured cell lines in either T175 cm^2^ or in multilayer culture flasks were rinsed three times with sterile Ca^++^ and Mg^++^ free PBS. The cells were dissociated by incubation with trypsin-free dissociation media (Sigma) for 10 min at 37 °C. They were flushed with PBS, recovered in a falcon tubes and harvested by 5 min centrifugation at 1000 g at 4 °C. The pellet was then resupended in a given volume of cold and 0.2 μm filtered PMCA buffer (Tris-HCl 50 mM pH 7.4, EDTA 5 mM, NaCl 300 mM, 1% Triton-X-100). The lysed cells were incubated at 4 °C during 15–30 min with gentle vortexing. The lysates were centrifuged at 2000 g during 6 min to pellet the insoluble and chromatin materials. Supernatants were collected, aliquoted and stored at −80 °C until use as substrate in Cell mb-PMCA reactions. Protein content of cell lysates was measured by Bradford protein concentration determination kit (BCA kit, Pierce) using BSA as standard.

### Cell-miniaturized beads-Protein Misfolding Cyclic Amplification (Cell-mb-PMCA)

The standard mb-PMCA, using brain lysate as source of PrP^C^ substrate was realized as described^22^, by using 96-well PCR microplates and one 2.384 mm teflon beads. The Cell-mb-PMCA was set up with either 100% cell lysate or a mix with 10% mouse PrP^0/0^ brain lysate (ratio 1:1) in the presence or absence of 1% of Dextran Sulfate Sodium (DSS > 500 kDa Sigma Aldrich, Saint Quentin Fallavier, France) as indicated. Practically, a 4 μl aliquot of the analyte inoculum (10^−n^ dilution) was suspended in 36 μl of PMCA substrate (brain or cell lysates) to obtain the 10^−n+1^ dilution. A series of 10-fold dilution was made by diluting 4 μL of the previous inoculum dilution to the next 36 μL containing well. Microplates were subjected to 96 cycles of 30 sec sonication at 200–220 Watt power (36–40% amplitude of the Q700 sonicators, Misonix, Farmingdale USA; or Delta Labo, Colombelles France) followed by 29.5 min of incubation at 37 °C. When needed, a second round of PMCA was realized with 1/10 diluted aliquot of the first round in fresh lysates. At the end of the PMCA, aliquots from each sample were analysed for PrP^res^ content by Western blotting.

### Protease digestion of PMCA products

To analyse the production of Proteinase K (PK)-resistant PrP^Sc^ species during PMCA, 10 μL of each sample were supplemented with SDS (up to 0.6% final concentration) and treated with PK (125 μg/mL final concentration) at 37 °C for 1 hour. The PK digestion was stopped by adding an equal volume of 2x Laemmli denaturation sample buffer and heating at 100 °C for 5 min. The samples were then stored at −20 °C. The levels of thermolysin-resistant PrP species in the PMCA amplicons were determined, as previously described^22^.

### SDS-PAGE and western blotting

PMCA samples were run on Criterion XT 12% Bis-Tris precast gels (Biorad, Hercules, CA, USA), electrotransferred onto nitrocellulose membranes with the semi-dry electrotransfer system (Biorad) and probed with biotinylated Sha31 anti-PrP monoclonal antibody^62^, as described above. PrP^C^ content of the cell lysates was determined by western blotting with SAF34^62^ anti octarepeat region of PrP. Quantification was determined with the GeneTools software after acquisition of the signals with a GeneGnome digital imager.

### Endpoint-titration of PMCA products in tg338 mice

Standard protocol based on the use of disposable equipment and preparation of all inocula in a class II microbiological safety cabinet was followed. Serial ten-fold dilutions of PMCA products were prepared in sterile 5% glucose containing 5% bovine serum albumin. Individually identified 6- to 10-week-old tg338 recipient mice (n = 5 mice per dilution) were inoculated intracerebrally with 20 μL of each sample. The inoculated animals were observed daily for the appearance of prion disease symptoms. Animals at terminal stage of disease were euthanized. The survival time was defined as the number of days from inoculation to euthanasia. Their brain and spleen were removed for PrP^res^ analysis by western blotting and histoblotting as previously described^22^,^33^. For histoblotting procedure, brains were rapidly removed from euthanized mice and frozen on dry ice. Cryosections were cut at 8–10 μm, transferred onto Superfrost slides and kept at −20 °C until use. Histoblot analyses were performed on 3 brains per dilution per amplicon, using the 12F10^63^ anti-PrP antibody.

To quantify vacuolar degeneration, brains were fixed in neutral-buffered 10% formalin (4% formaldehyde) before paraffin embedding. After deparaffinization, 2-μm-thick sections were stained with hematoxylin-eosin. Vacuolation profiles were established according to the standard method described by Fraser and Dickinson^64^, using three brains per experiment.

## Additional Information

**How to cite this article**: Moudjou, M. *et al*. Glycoform-independent prion conversion by highly efficient, cell-based, protein misfolding cyclic amplification. *Sci. Rep.* **6**, 29116; doi: 10.1038/srep29116 (2016).

## Supplementary Material

Supplementary Information

## Figures and Tables

**Table 1:** Incubation time of tg338 mice inoculated with serial tenfold dilutions of Cell-based mb-PMCA-generated 127S prions.

Dilution	Incubation time in days ± SEM (n/n_0_)		
	PMCA substrate		
	Cell lysate − DSS	Cell lysate + DSS	*tg338 brain*
10^−1^	75 ± 1 (5/5)	93 ± 1 (5/5)	*60* ± *1 (4/4)*
10^−3^	91 ± 2 (5/5)	100 ± 4 (5/5)	*72* ± *2 (5/5)*
10^−4^		108 ± 6 (5/5)	*ND*
10^−5^	114 (1/5)*	98; 124 (2/5)*	*98* ± *3 (5/5)*
10^−6^		>240 (0/5)*	*ND*
10^−7^	>240 (0/5)*	>240 (0/5)*	*124 (1/5)*
Unseeded	>240 (0/5)*	>240 (0/5)*	

Amplicons obtained from a 10^−7^ 127S seed mixed with 10 mg/mL cell lysates supplemented with PrP^0/0^ brain, in the absence (−DSS) or presence (+DSS) of 1% dextran sulfate, were tenfold diluted up to the 10^−7^ dilution and immediately inoculated intracerebrally to reporter tg338 mice. n/n_0_: number of mice with neurological disease and positive for PrP^res^ in the brain by immunoblotting/number of inoculated tg338 mice.

^*^Non-affected mice euthanized healthy at 240 dpi. Data in italic are from^22^. ND: not done.

**Table 2:** Convertibility of the PrP glycosylation mutants in cell culture and by cell PMCA.

Glycotype	Genotype	Cell culture conversion*	Cell-PMCA conversion	Incubation time of tg338 mice inoculated with Cell-PMCA amplicons in days ± SEM (n/n_0_)
Wild type (VRQ)	Wild type	+	+	70 ± 1 (5/5)
Monoglycosylated (Site 1)	N184D	+	+	68 ± 1 (5/5)
	N184Q	_	+	77 ± 1 (5/5)
Monoglycosylated (Site 2)	N200D	+	+	69 ± 1 (5/5)
	N200Q	+	+	nd
Unglysosylated	N184D-N200D	−	+	68 ± 1 (5/5)
Unglysosylated mutants → tg338 brain PMCA#			+	65 ± 1 (5/5)
Unseeded Wild type (VRQ)	none		−	>250 (0/5)

n/n_0_: number of mice with neurological disease and positive for PrP^res^ in the brain by immunoblotting/number of inoculated tg338 mice. The seeds used to infect mice were from a 127S 10^−7^ seed amplified over 2 rounds, so as to avoid any residual input.

^*^Cell conversion was assessed in ref. 14.

^#^The PMCA product inoculated was obtained by seeding tg338 brain lysate with unglycosylated PrP^Sc^ seed (10^−7^ dilution), itself obtained by seeding the NDND double mutant cell lysate with 10^−8^ diluted 127S seed. nd: not done.
