# Supplementary material for: Glycoform-independent prion conversion by highly efficient, cell-based, protein misfolding cyclic amplification
Source: Sci Rep. 2016 Jul 7;6:29116. doi: 10.1038/srep29116 (PMC4935985; doi:10.1038/srep29116)
Supplement: Supplementary Information [file srep29116-s1.doc]

**Supplementary Information**

**Glycoform-independent prion conversion by highly efficient, cell-based, protein misfolding cyclic amplification**

Mohammed Moudjou^1^*^†^, Jérôme Chapuis^1^*, Mériem Mekrouti^1^, Fabienne Reine^1^, Laetitia Herzog^1^, Pierre Sibille^1^, Hubert Laude^1^, Didier Vilette^1,2^, Olivier Andréoletti^2^, Human Rezaei^1^, Michel Dron^1^ and Vincent Béringue^1 †^

^1^VIM, INRA, Université Paris-Saclay, 78350, Jouy-en-Josas, France

*^2^*IHAP, INRA, Ecole Nationale Vétérinaire de Toulouse, 31000, Toulouse, France.

Correspondence: vincent.beringue@jouy.inra.fr; mohammed.moudjou@jouy.inra.fr

^†^Share senior authorship

*Equal contributors

**Inventory of Supplementary Information**

Figure S1

Figure S2

Figure S3


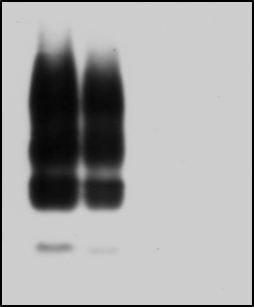


**38**

**28**

**17**

**P2FJ6**

**RK13**

**-5 -6 -5 -6**

**Log_10_**

**(inoculum dilution)**

**R1 R2 R1 R2**

**Figure S1. No PMCA amplification of 127S prions with the parental RK13 cells.**

Lysates from RK13 cells expressing (P2FJ6) or not (RK13) ovine PrP^C^ were used as PMCA substrate to amplify 10^5^-fold diluted 127S prions over two rounds (R). Each round was analysed by western blotting for PrP^res^ content (Sha31 antibody).


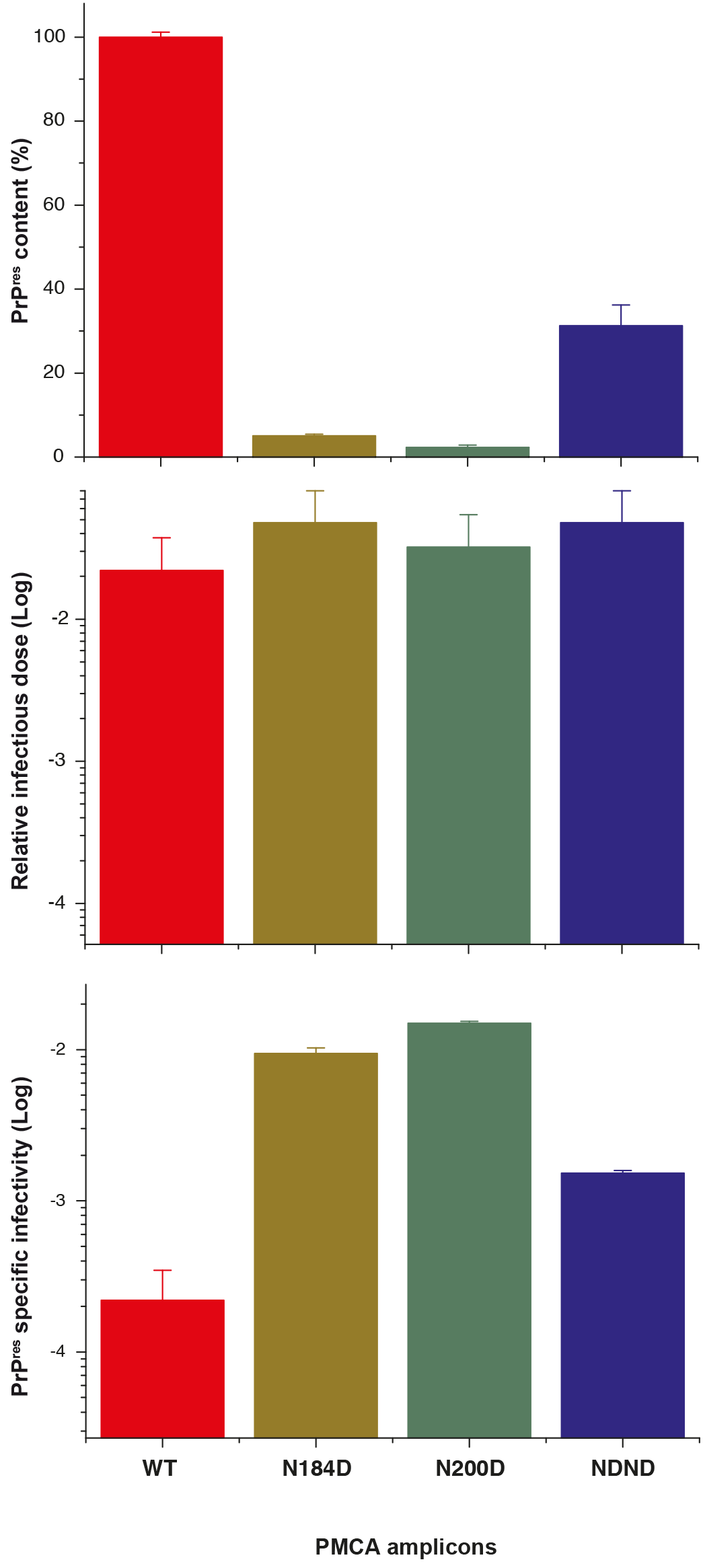


**Figure S2. Specific infectivity per unit PrP^res^ of the PMCA amplicons.**

The PMCA products generated with cells lysates from RK13 cells expressing ovine PrP^C^ mutated on the first N-glycosylation site at residue 184 (N184D), on the second glycosylation site at residue 200 (N200D, N200Q) or at both residue (NDND) were quantified by western blot for PrP^res^ content (n=3 independent PMCA, round 2). These products were inoculated to reporter tg338 mice for infectivity content. Applying the mean survival times values (see **Table 2**) to standard dose response curve for 127S prions^1^ allowed calculating the relative infectious dose per amplicon. A relative infectious dose of 0 corresponds to animal inoculated with the 2 mg equivalent of infectious tg338 brain tissue. The specific infectivity per unit PrP^res^ was determined by dividing these values by the amount of PrP^res^ per amplicon.


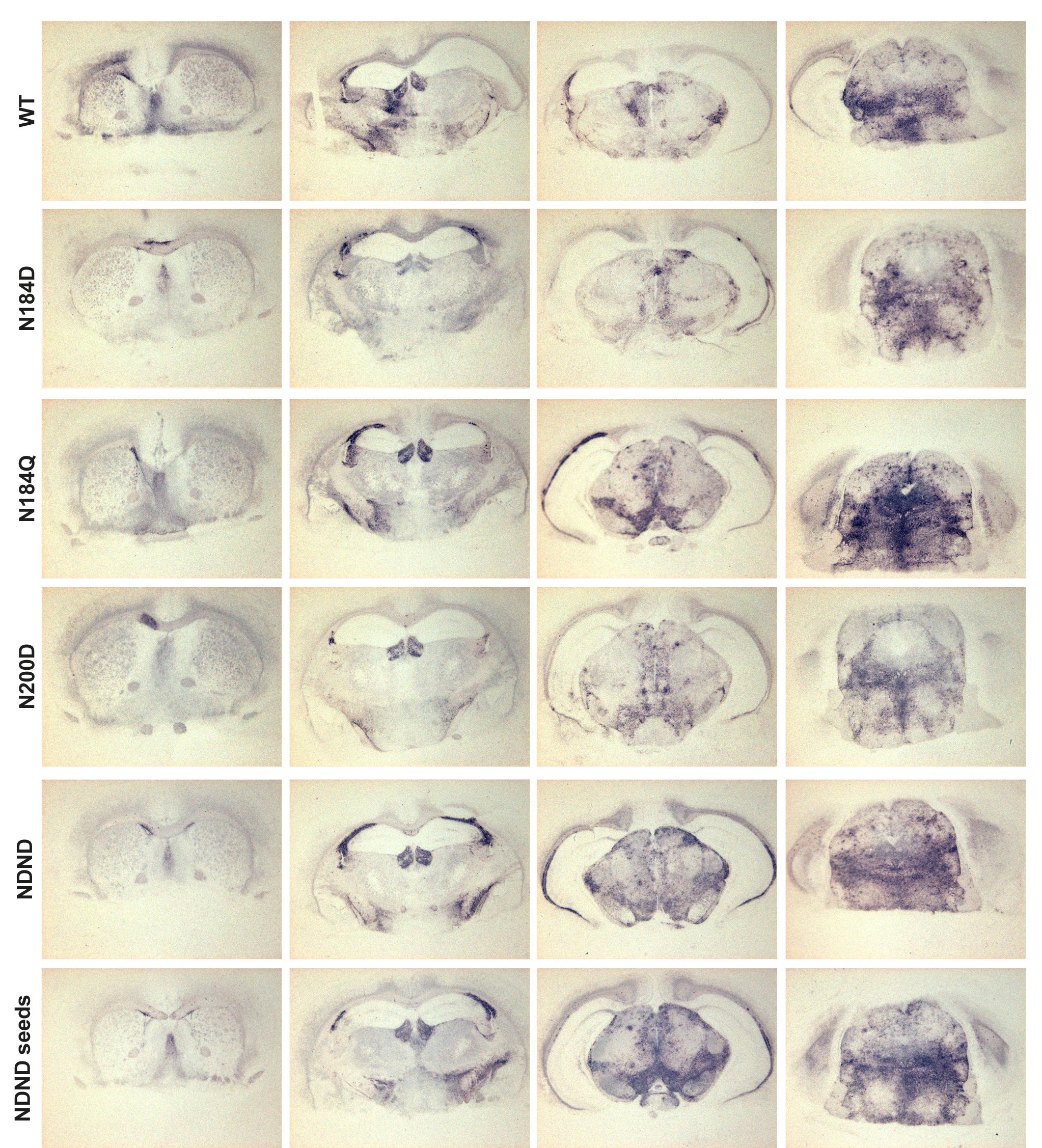


**Figure S3. PrPres deposition in the brains of tg338 mice inoculated with Cell-mb-PMCA lowly-glycosylated 127S prions.**

Lysates from RK13 cells expressing wild-type (WT) ovine PrP^C^ or ovine PrP^C^ mutated on the first N-glycosylation site at residue 184 (N184D, N184Q), on the second glycosylation site at residue 200 (N200D) or at both residue (NDND) glycosylation sites were mixed with PrP^0/0^ brain lysate (1:1 dilution), seeded with serial 10-fold dilutions of tg338 brain homogenate containing 127S prions and submitted to 2 rounds of Cell-mb-PMCA before inoculation to tg338 mice. Seeds generated from NDND cells were also submitted to another round of PMCA using tg338 mouse brain as substrate (wild type brain PrP^C^). The amplicon obtained at 10^-8^ dilution was then used for inoculation (NDND seeds). Representative histoblots of antero-posterior coronal brain sections (12F10 antibody) at the level of the septum, hippocampus, midbrain and brainstem (from left to right). The pattern observed is specific of 127S, with prominent deposition in the corpus callosum, lateral hypothalamic nuclei and raphe nuclei of the brain stem.

**References**

1 Tixador, *P. et a*l. The physical relationship between infectivity and prion protein aggregates is strain-dependent*. PLoS Path*o**g** 6, e1000859, doi:10.1371/journal.ppat.1000859 (2010).
